# Applications and advances in molecular diagnostics: revolutionizing non-tuberculous mycobacteria species and subspecies identification

**DOI:** 10.3389/fpubh.2024.1410672

**Published:** 2024-06-19

**Authors:** Haiyang Zhang, Maoting Tang, Deyuan Li, Min Xu, Yusen Ao, Liangkang Lin

**Affiliations:** ^1^Department of Pediatrics, West China Second University Hospital, Sichuan University, Chengdu, China; ^2^Key Laboratory of Birth Defects and Related Diseases of Women and Children, Sichuan University, Ministry of Education, Chengdu, China; ^3^Department of Pediatrics, The Eighth Affiliated Hospital, Sun Yat-sen University, Shenzhen, China

**Keywords:** non-tuberculous mycobacterium, molecular diagnostics, whole genome sequencing, next-generation sequencing, drug resistance gene

## Abstract

Non-tuberculous mycobacteria (NTM) infections pose a significant public health challenge worldwide, affecting individuals across a wide spectrum of immune statuses. Recent epidemiological studies indicate rising incidence rates in both immunocompromised and immunocompetent populations, underscoring the need for enhanced diagnostic and therapeutic approaches. NTM infections often present with symptoms similar to those of tuberculosis, yet with less specificity, increasing the risk of misdiagnosis and potentially adverse outcomes for patients. Consequently, rapid and accurate identification of the pathogen is crucial for precise diagnosis and treatment. Traditional detection methods, notably microbiological culture, are hampered by lengthy incubation periods and a limited capacity to differentiate closely related NTM subtypes, thereby delaying diagnosis and the initiation of targeted therapies. Emerging diagnostic technologies offer new possibilities for the swift detection and accurate identification of NTM infections, playing a critical role in early diagnosis and providing more accurate and comprehensive information. This review delineates the current molecular methodologies for NTM species and subspecies identification. We critically assess the limitations and challenges inherent in these technologies for diagnosing NTM and explore potential future directions for their advancement. It aims to provide valuable insights into advancing the application of molecular diagnostic techniques in NTM infection identification.

## 1 Introduction

Non-tuberculous mycobacteria (NTM) encompass a diverse assembly of species distinct from the *Mycobacterium tuberculosis* complex (MTBC) and *Mycobacterium leprae* (*M. leprae*). Identified initially in the late 19th century, NTM are characterized as Gram-positive, rod-shaped bacteria, often exhibiting branching or curved formations. The cell wall structure of NTM, rich in lipids, phenolic glycolipids, and mycolic acids, is a key biological marker, rendering these bacteria acid-fast positive, similar to *Mycobacterium tuberculosis* (MTB), as demonstrated by staining techniques like the Ziehl-Neelsen staining method (1, 2). However, while useful, acid-fast staining alone cannot distinguish between MTB and NTM, necessitating further molecular diagnostic interventions for accurate identification. NTM is classified based on their growth rate in subculture as either rapidly growing mycobacteria (RGM), with a growth cycle of less than 7 days, or slowly growing mycobacteria (SGM), requiring 7 days or more to grow. To date, approximately 200 species of NTM have been identified, which can be further differentiated into various subspecies. The distinct clinical features and treatment responses of different subspecies play a crucial role in understanding the clinical management and therapeutic strategies for diseases associated with NTM (3, 4). The species diversity of NTM significantly varies across global regions, with the pathogenic potential of NTM species being geographically distinct. Additionally, in developed nations, the disease burden attributable to NTM infections now exceeds that of MTB (5–8).

NTM infections can manifest across multiple tissue and organ systems, such as the respiratory, central nervous, lymphatic, articular, and dermal systems. Notably, pulmonary infections account for roughly 90% of these cases (9, 10). The clinical manifestations of pulmonary infections caused by NTM are characterized by their nonspecific nature. Although chronic or recurrent coughing is almost universally reported among patients, there is variability in symptoms such as expectoration, fatigue, malaise, dyspnea, fever, hemoptysis, chest pain, and weight loss. In children aged 1 to 5 years, NTM primarily infects submandibular, cervical, or preauricular lymph nodes, indicating its capacity to also target tissues beyond the commonly affected systems (11, 12). Currently, approximately 80% of culture-confirmed NTM lymphadenitis cases are caused by the *Mycobacterium avium* complex (MAC) (13). Recent studies emphasize the evolving landscape of NTM species affecting lymph nodes, highlighting the need for advanced molecular diagnostics to accurately identify and tailor treatment strategies. Furthermore, studies reported a steady increase in the variety of NTM species isolated from lymph nodes, including *Mycobacterium lentiflavum* (*M. lentiflavum*) (14).

Accurate diagnosis and treatment of NTM infections necessitate a nuanced and individualized assessment of therapeutic risks versus benefits. The widespread environmental presence of NTM poses substantial challenges for clinicians in accurately identifying infections. Detecting NTM in non-sterile respiratory specimens does not necessarily indicate pathogenic involvement in pulmonary conditions, potentially indicating colonization, transient infection without clinical disease, or sample contamination. Furthermore, the pathogenic potential of NTM significantly depends on the host’s immune status and the specific anatomical site of culture extraction. When NTM is isolated from sterile sites, including blood, tissues, cerebrospinal fluid, pleural fluid, or the brain, its presence almost always indicates clinical significance (15).

## 2 The critical role of molecular diagnostic techniques in enhancing the precision and efficiency of NTM species and subspecies identification

Traditionally, the identification of species within the NTM complex has relied on time-consuming biochemical tests and methods based on phenotypic characteristics, which often fail to provide accurate identification results. Pathogenic culture, despite its limitations, has historically been the gold standard for NTM identification and remains critical for drug sensitivity testing (DST) (16). While culture is indispensable for DST, molecular diagnostics play an increasingly crucial role in genotypic identification, offering more rapid and accurate results (4). The effectiveness of culture-based identification is markedly impacted by the growth kinetics of mycobacteria. Specifically, slow-growing strains of NTM often necessitate upwards of 7 days to yield discernible colonies. This contrasts sharply with molecular techniques, which can often identify NTM species within hours. Additionally, specific NTM species and subspecies, such as *Mycobacterium haemophilum* (*M. haemophilum*), *Mycobacterium marinum* (*M. marinum*), *Mycobacterium ulcerans* (*M. ulcerans*), and MAC, often cannot be identified through conventional culturing methods due to their stringent dependency on certain nutrients or specific culturing conditions (17–19).

To address these challenges, advancements in molecular diagnostics have revolutionized the identification of NTM infections, offering substantial improvements over traditional methods in terms of accuracy, diagnostic sensitivity, speed, and cost-effectiveness, especially in differentiating NTM species and genotyping. Several detection methods for NTM have been developed by adapting techniques originally designed for mycobacterial identification. While targeted single-gene sequencing effectively identifies most NTM species, the distinction at the subspecies level often requires multigene sequencing approaches (20, 21). Table 1 summarizes the comparison of DNA-based molecular techniques for identification of genetically closa NTM species, underscoring the critical role of these diagnostics in the precise detection of NTM (22–54).

**Table 1:** Comparison of DNA-based molecular techniques for identification of genetically closa NTM species.

Methods			Throughput	Resolution	Sensitivity	Specificity	Depth	Advantage(s)	Limitations	References
Repetitive sequence-based methods	MLVA	MIRU-VNTR	Poor	High	84.8% in *Mycobacterium ulcerans*	High	subspecies level	Fast, easy to perform, sensitive, highly reproducible, and discriminative; more discriminatory than IS6110- RFLP for NTM isolates with low copy no. Of IS6110; well suited for largescale, genetic, or evolutionary investigations; digitized results; can be performed directly on cell lysates; applicable for typing of NTM	The impact of VNTR marker choice, absence of uniform standards, the essentiality of advanced bioinformatics expertise and analytical instruments, and the need for high-integrity DNA.	(22–27)
	Rep-PCR		Poor	Moderate	Moderate	High	subspecies level	Commercially available;highthroughput automated system for typing of many NTM species; achieves higher level of discrimination than MIRU-VNTR typing for *M. avium* and provides better reproducibility	Necessity for process refinement and establishment of standard protocols, requirement for comprehensive bioinformatics expertise and advanced analytical technology, associated with significant expenditure.	(27–32)
Non-repetitive sequence-based techniques	Gene sequence analysis	Hybridization Probes	Poor	High	High	High	subspecies level	This method does not require a PCR amplification step and enables rapid detection and diagnosis	Relies on specific primers, limiting its applicability across diverse bacterial species.The clinically important *Mycobacterium abscessus* could not be identified	(33–35)
		LPA	Poor	High	High	High	subspecies level	This method has a low cost and no need to purchase special equipment	Limitations may arise due to incomplete coverage of NTM species, potential cross-reactivity, and the requirements for specialized equipment and technical expertise.	(36, 37)
		DNA microarrays	High	High	98.8%	100%	subspecies level	The detection method has fast detection speed, accurate results, high throughput and high degree of automation	Relies on specific primers, limiting its applicability across diverse bacterial species.	(38)
		REBA	Poor	High	87.61%	83.33%	subspecies level	The assay has high sensitivity, specificity and stability	Relies on specific primers, limiting its applicability across diverse bacterial species.	(39)
		PCR-RFLP	Poor	Moderate	High	Moderate	subspecies level	Does not require specialized equipment	Subject to the limitations of primers and restriction enzymes, this method involves tedious procedures, is restricted by enzyme recognition sites, and necessitates high-quality DNA.	(27, 40–42)
	Genome analysis	PFGE	High	High	High	Moderate	subspecies level	Inexpensive; data analysis easier than with REA	Characterized by significant equipment and technical demands, this method is expensive, time-intensive, not suited for high-throughput applications, and needs high-quality DNA.	(27, 43–45)
		RAPD	Moderate	High	Moderate	Moderate	subspecies level	Can be performed on unknown DNA sequence	Suffers from low resolution, inconsistent repeatability, reduced specificity, reliance on primers, and difficulty in interpreting outcomes.	(27, 46)
		AFLP	High	High	Moderate	High	subspecies level	Broad range of possible adjustments to improve discriminatory power of the method	Involves intricate procedures, is expensive, dependent on primers, requires complicated data analysis, and is difficult to standardize.	(27, 47, 48)
		LSP	High	High	Moderate	Moderate	subspecies level	Deligotyping is a very sensitive and efficacious approach for rapid screening of clinical isolates of NTM. The method is also well suited for constructing robust phylogenetic relationships	Dependent on the availability of a reference genome, limited applicability to various strains, and requires high-quality DNA.	(22, 49)
		WGS	High	High	High	High	subspecies level	May be performed directly on clinical samples (metagenomic approach); provides information on (nearly) the entire genome; allows detection of different genetic variants within the same population	Demands advanced equipment and technical proficiency, involves intricate data processing and analysis, is expensive, time-intensive, and necessitates high-quality DNA.	(27, 36, 50, 51)
	NGS	mNGS	High	High	High	High	subspecies level	Can be performed on unknown DNA sequence, Detects previously unknown mutations	Characterized by significant equipment and technical demands, this method involves complex data processing and analysis, is expensive, and exhibits low sensitivity for detecting target pathogens.	(52–54)
		tNGS	High	High	High	High	subspecies level	This assay has high throughput, strong targeting, low detection cost, and targeted detection of known pathogen subtypes and different drug resistance genes	Involves complicated data processing and analysis, is expensive, time-intensive, incapable of detecting novel pathogens, and necessitates a well-defined reference genome for probe design.	(53, 54)

MLVA, multilocus variable number of tandem repeat analysis; MIRU-VNTR, mycobacterial interspersed repetitive unit-variable number tandem repeat; Rep-PCR, repetitive element palindromic PCR; LPA, line probe assay; REBA, reverse blot hybridization assay; PCR-RFLP, PCR-restriction fragment length polymorphism; PFGE, pulsed-field gel electrophoresis; RAPD, randomly amplified polymorphic DNA; AFLP, amplified fragment length polymorphism; LSP, large sequence polymorphism; WGS, whole genome sequencing; mNGS, metagenomic next-generation sequencing; tNGS, targeted next-generation sequencing.

## 3 Repetitive sequence-based methods in NTM identification

Repetitive sequence-based methods are widely utilized in molecular biology for the accurate identification of NTM. Leveraging the substantial variability inherent in the repetitive regions of bacterial genomes, these techniques distinguish between strains through comparative analysis of sequence differences.

### 3.1 Repetitive sequences involving NTM

Insertion sequence (IS) is a compact, mobile genetic element that predominantly encodes for transposition and regulatory functions. Its integration into specific genomic loci can disrupt gene functionality and alter the expression of nearby genes. Investigations across various NTM species have revealed multiple distinct IS types, underscoring their pivotal role in molecular diagnostics and contributing to genomic diversity. Among these IS elements, IS*1245* and IS*1311* are closely associated with the molecular typing of MAC strains (55). In addition to IS*1245* and IS*1311*, the MAC genome includes other insertion sequences. These insertion elements include IS*900*, present in *M. avium* subsp. *paratuberculosis* (MAP), IS*901* in *M. avium* subsp. a*vium* (MAA), IS*902* in *M. avium* subsp. s*ilvaticum* (MAS), and those yet to be thoroughly investigated in *M. avium* isolates (21, 56, 57). IS*900*, IS*901*, and IS*902*, of the reported IS elements thus far, have been widely used to identify and differentiate various MAC strains (58–60). Extending **b**eyond MAC, there are numerous IS used for NTM epidemiological studies, highlighting the expansive utility of IS in the broader context of NTM research. These IS include IS*1395* in *M. xenopi*, IS*1511*/IS*1512* in *Mycobacterium gordonae* (*M. gordonae*), IS*1407* in *M. celatum*, IS*6120* and IS*2404* in *Mycobacterium smegmatis* (*M. smegmatis*), IS*2606* in *M. ulcerans*, *M. lentiflavum*, and *Mycobacterium kansasii* (*M. kansasii*), as well as IS*1652* in *M. kansasii* (61–66).

Trinucleotide repeat sequence (TRS) is ubiquitously present across bacterial genomes, varying in quantity, and serve as a pivotal tool for bacterial genotyping. Within the genomes of mycobacteria, the majority of these TRS are located within the genes of the Pro-Glu (PE) and PPE families (67). The PGRS-restriction fragment length polymorphism (RFLP) typing technique, which relies on the PGRS sequences, involves cloning these sequences into the recombinant plasmid pTBN12 to serve as probes for species identification. This approach has identified PGRS sequences across various NTM species, thereby facilitating the differentiation of strains such as *M. kansasii* and *M. ulcerans* through similar PGRS-RFLP typing methods. This advancement underscores the utility of TRS in the molecular diagnostics landscape, significantly enhancing the resolution of mycobacterial genotyping and contributing to our understanding of NTM infection identification (21).

Enterobacterial repetitive intergenic consensus (ERIC), characterized as imperfect palindromic sequences of approximately 126 bp, are predominantly distributed within the genomes of Gram-negative bacteria. The variability of ERIC sequences has facilitated the development of a novel genotyping approach, known as enterobacterial repetitive intergenic consensus-polymerase chain reaction (ERIC-PCR) typing (68). ERIC, a derivative of the RAPD technique, proves to be informative when utilized for targets beyond *Enterobacteria* (69). This method has been extensively applied to assess the genetic diversity among various species of Mycobacteria, including MTB, *M. gordonae*, *Mycobacterium intracellulare* (*M. intracellulare*), *Mycobacterium szulgai* (*M. szulgai*), *Mycobacterium fortuitum* (*M. fortuitum*), *Mycobacterium chelonae* (*M. chelonae*), and *Mycobacterium abscessus* (*M. abscessus*) (70–73). ERIC-PCR typing has significantly enhanced insights into the molecular epidemiology and phylogenetic relationships of these species, improving our comprehension of their transmission dynamics and pathogenic potential (74).

### 3.2 Repetitive sequence-based methods

Multilocus variable number of tandem repeat analysis (MLVA) characterizes tandem repeat regions dispersed across the NTM genome. These regions mirror the polymorphic minisatellites found in eukaryotic genomes. The variable number of tandem repeat (VNTR) loci encompasses five major polymorphic tandem repeat (MPTR) sequences (MPTR-A to E) and six exact tandem repeat (ETR) sequences (ETR-A to F) (75). MPTR sequences, comprising unique 10 bp sequences separated by 5 bp intervals, are identified across various NTM species, including *M. gordonae*, *M. kansasii*, and *M. szulgai* (76). MPTR sequences have been demonstrated to facilitate RFLP typing of *M. kansasii*, utilizing MPTR sequences as probes (66, 77). Supply et al. (78) introduced an optimized 24-locus mycobacterial interspersed repetitive units-variable number tandem repeats (MIRU-VNTR) typing scheme, incorporating 12 loci previously identified. This expanded 24-locus format offers enhanced phylogenetic insight, establishing it as a standard method for typing the MTBC. MIRU-VNTR analysis is extensively applied in NTM typing, notably within *M. avium*, where the identification of multiple loci has revealed substantial discriminatory capability. In the case of *M. intracellulare*, up to 45 potential loci have been identified, with 7 exhibiting high variability, making them suitable for differentiation purposes. Additionally, 13 loci have been employed in the study of isolated *M. ulcerans*, further evidencing the utility of MIRU-VNTR analysis in nuanced differentiation and identification (24, 79).

Repetitive element palindromic PCR (Rep-PCR) represents a commercialized, high-throughput, automated system designed to genotype various species of mycobacteria by leveraging the variability in repetitive sequences scattered across bacterial genomes. This method amplifies repetitive fragments within non-coding sequences and separates them using a microfluidic electrophoresis chip (28). The NTM genome is characterized by various repetitive elements, including IS*6110*. The variability in sequence copy numbers and configurations yields unique genomic fingerprints. Rep-PCR leverages this to generate distinct profiles, differentiating closely related species or strains via subtle pattern shifts. Although some NTM species conserve specific repeating elements, others exhibit significant variations. This combination of conservation and variability aids in identifying NTM at both the species and strain levels with precision. Rep-PCR has been applied to the genotyping of *M. abscessus*, and its typing patterns have been compared with those obtained via pulsed-field gel electrophoresis (PFGE). The results demonstrated a 90% concordance in the typing of identical strains between Rep-PCR and PFGE analyses, suggesting that Rep-PCR may offer superior discriminatory power over PFGE (80). In the typing of various NTM species, Rep-PCR demonstrates enhanced repeatability relative to random amplified polymorphic DNA (RAPD) analysis. Furthermore, Rep-PCR offers superior discriminatory power compared to MIRU-VNTR typing for the MAC, underscoring its potential to improve resolution in NTM infection molecular diagnostics (72, 81).

## 4 Non-repetitive sequence-based methods in NTM identification

Non-repetitive sequence-based methods, while serving as a cornerstone in genetics, evolutionary biology, and biological research, have also been increasingly adopted in the molecular diagnostics of infectious diseases, including the identification of NTM infections. These methods facilitate the precise differentiation of NTM species, which are crucial for tailored therapeutic strategies. Contrary to repetitive sequences, non-repetitive sequences encompass gene-coding regions, regulatory elements, and functional sequences that, although not as prevalent as repetitive elements in the genome, play pivotal roles in genetic diversity and function. The analysis of non-repetitive sequences, through sequence alignment and specific gene or fragment sequencing, is pivotal for detecting genetic variations within NTM species.

### 4.1 Gene sequence analysis

Gene sequence analysis stands as a pivotal bioinformatics approach for examining and decoding the DNA sequences within genomes, playing an essential role in the identification and study of NTM infections. By leveraging computational algorithms and bioinformatics tools for DNA sequence alignment, assembly, annotation, and analysis, this approach elucidates the NTM genome’s structure, function, and evolution. It is instrumental in accurately distinguishing NTM species and subspecies, essential for precise treatment and management. Furthermore, genomic insights not only enhance our understanding of NTM biodiversity but also illuminate its public health implications, including the emerging patterns of antibiotic resistance and the geographical distribution of infections. This knowledge is imperative for informing public health strategies and interventions aimed at preventing and managing NTM outbreaks. Single nucleotide polymorphism (SNP) typing has shown low levels of homogeneity, making it a valuable tool for differentiating between species and thus has been employed in the identification of mycobacterial species. Technological advancements now enable the use of molecular beacons to identify single nucleotide substitutions, facilitating simultaneous analysis of multiple SNP loci. Gene sequence analysis leveraging SNP typing facilitates the identification of a broad spectrum of mycobacterial species, encompassing members within the MTBC, and enables the detection of various resistance markers (21, 82). Gene sequence analysis methods yield better results with the analysis of multiple loci. Multilocus sequence typing (MLST), which involves sequencing allele groups to differentiate species, is extended significantly in mycobacterial identification. Tools like PubMLST and mlstverse cater to the nuanced demand for subspecies-level identification (83, 84). PubMLST, combining conventional and ribosomal MLST, facilitates comprehensive bacterial identification. By accumulating and integrating genomic sequences from MLST, enhanced typing can be linked to prognosis and treatment resistance in emerging subspecies. This approach has effectively distinguished members of the MAC and fast-growing mycobacteria, although some studies indicate that MLST alone may not suffice for precise strain differentiation (43, 55, 85).

Initially, the utilization of DNA probe-based techniques to identify partial gene fragments represented a common technique in molecular biology. Depending on the type of target nucleic acid, a variety of amplification methods are employed based on their specific advantages, including conventional PCR for its simplicity and cost-effectiveness, real-time PCR for its quantitative capabilities, nucleic acid sequence-based amplification for high sensitivity, loop-mediated isothermal amplification for rapid and equipment-free amplification, and transcription-mediated amplification for its high amplification efficiency (86–90). Gene probe assays are available in two primary variants. Direct nucleic acid testing (NAT) traditionally required a substantial amount of bacterial material, although recent advancements in sensitivity allow for effective analysis with reduced bacterial loads. Incorporating an amplification step in nucleic acid amplification tests (NAATs) allows for the direct detection of bacterial DNA in clinical samples. The market offers a diverse array of commercial nucleic acid probes, which, when combined with the aforementioned amplification methods, enhance the detection and differentiation capabilities for a wide range of mycobacteria (encompassing both MTBC and NTM), with target sequences often including rRNA, 16S rRNA, and the 16S-23S rRNA spacer region (65, 91, 92). The line probe assay (LPA) employs a technique where target DNA is amplified using biotin-labeled specific primers. Subsequently, the amplified product is denatured and hybridized with specific oligonucleotides affixed to a nylon membrane. The results are then visualized through enzyme-linked immunochromatography. Notable LPA kits include the INNO-LiPA Mycobacteria v2 (Fujirebio Europe, Belgium), GenoType Mycobacteria CM, GenoType Mycobacteria AS, and GenoType NTM-DR kits (Hain Life Sciences, Germany), along with the Speed-oligo Mycobacteria (Vircell, Spain). The GenoType NTM-DR kits not only facilitates the identification of commonly encountered NTM to the species level, such as MAC, *Mycobacterium abscessus* complex (MABC), and *M. chelonae*, but also enables the detection of resistance to macrolides. This is achieved through the identification of mutations in the *erm41* gene and *rrl* gene, as well as resistance to aminoglycosides by detecting mutations in the *rrs* gene (37). However, in clinical mycobacterial identification laboratories, many still rely on commercial single-stranded DNA nucleic acid probe technology. This approach typically offers high species identification accuracy, with results available in a short timeframe (less than 24 h), facilitating rapid identification (93). The reverse blot hybridization assay (REBA) operates on the principle of affixing numbered oligonucleotide probes to a nitrocellulose or nylon membrane. These probes are then hybridized with biotin-labeled PCR amplification products. The presence of a colored signal at specific positions on the membrane strip indicates successful hybridization of the probe to the DNA fragment. Wang HY et al. conducted a comparative analysis of PCR-REBA with real-time PCR and RFLP techniques (94). Their findings demonstrated that PCR-REBA delivers highly sensitive and specific results in identifying NTM and distinguishing between NTM species from mycobacterial liquid cultures. DNA microarrays present a compelling alternative for conducting high-throughput analyses of multiple genetic markers concurrently, distinguishing themselves from traditional methods reliant on predefined probes. These chips can be tailored to target specific regions of interest within bacterial genomes, including conserved sequences typically associated with NTM species. This customization enables a more exhaustive analysis, facilitating the detection of genetic variations and the differentiation of closely related species. Despite the potential for elevated costs, the versatility and efficiency of DNA microarrays are increasingly appealing to clinical laboratories specializing in mycobacterium identification. Research has explored the utility of DNA microarrays in NTM identification (95, 96). With ongoing technological advancements driving down costs and enhancing performance, DNA microarrays are poised to become indispensable tools for NTM monitoring and research, addressing critical challenges in clinical settings and aiding in the development of more effective management strategies.

Currently, PCR-RFLP has been effectively employed in the identification of NTM, marking a significant advancement in gene sequence analysis. This method integrates PCR amplification, restriction enzyme digestion, and electrophoresis to generate species or strain-specific profiles. For the identification of NTM species using this approach, the process begins with the PCR amplification of the *rpoB* gene and the *hsp65* gene, resulting in PCR products. These are then subjected to digestion with specific restriction enzymes, such as MspI, HaeIII, and BstEII, followed by analysis using agarose gel electrophoresis. By employing additional restriction enzymes, this technique can further differentiate members of the MABC into subspecies levels (97).

### 4.2 Genome analysis

Genome analysis transcends basic gene sequencing, offering a detailed exploration of an organism’s full genome, including gene organization, structure, and overall genomic context. This inclusive strategy provides intricate, high-resolution insights into both coding and non-coding regions and regulatory elements, shedding light on genetic variations, evolutionary links, and functional attributes. Consequently, it delivers a nuanced understanding of strain characteristics and their interconnections.

PFGE uses restriction enzyme digestion to fragment chromosomal DNA from different mycobacterial strains, producing unique fingerprint patterns that are visible under ultraviolet light after gel electrophoresis. The technique applies periodically alternating electric fields to direct DNA fragments, facilitating the separation of large molecules more effectively. A principal advantage is its capacity to separate larger DNA fragments, beyond the 50 kb limit of conventional unidirectional electrophoresis, by employing rare-cutting restriction enzymes. PFGE is characterized by its high repeatability and discriminatory capacity. Despite subtle strain variabilities, it proves highly effective in typing diverse NTM species, demonstrating particular success with slow-growing strains such as *M. kansasii*, MAC, and *M. avium* (98). However, it’s important to acknowledge that PFGE involves significant costs, demands extensive technical expertise, and has a lengthy turnaround time, sometimes extending to 5 days. Like RFLP, PFGE also necessitates access to a comprehensive database of high-quality DNA sequences for reference (99).

Randomly amplified polymorphic DNA (RAPD), or arbitrary primed PCR (AP-PCR), is a technique independent of prior DNA sequence knowledge. Utilizing a single, arbitrarily chosen primer of 5 to 50 bp, it generates strain-specific DNA profiles by binding at sites with full or partial template DNA matches (100). Despite its high discriminative power, RAPD is limited by its poor reproducibility. Furthermore, it is generally believed that the observed differences between strains are more attributable to technical and procedural variations inherent to the method rather than true genetic polymorphism (101). Despite its limitations, RAPD-especially with multiple primer combinations-has been effectively used for NTM strain analysis. The polymorphism of DNA profiles from these combinations matches or exceeds those from PFGE. Moreover, RAPD’s application extends to genotyping *M. abscessus* and *M. chelonae*, which are prone to spontaneous fragmentation during gel electrophoresis, thus complicating PFGE assessment (102, 103). Similarly, this method has been used for typing strains of *Mycobacterium phocaicum* (*M. phocaicum*), *M. gordonae*, *M. szulgai*, and *Mycobacterium malmoense* (*M. malmoense*) (104–107).

Amplified fragment length polymorphism (AFLP) analysis represents a PCR-based genotyping technique that utilizes dual restriction enzymes for DNA digestion: one rare cutter and one frequent cutter, recognizing sites of 6 bp and 4 bp, respectively. Following digestion, the resultant DNA fragments are ligated to double-stranded adaptors ranging from 10 to 30 bp, which are complementary to PCR primers. This setup facilitates the selective amplification of fragment subsets. AFLP analysis has emerged as a novel approach for typing NTM species, including members of the MAC and *M. hemophilum* (27). Additionally, AFLP has proven effective in distinguishing between closely related species, such as *M. marinum* and *M. ulcerans*, offering a refined tool for microbial identification and strain differentiation within the context of molecular diagnostics (21).

Large sequence polymorphism (LSP) analysis, a key molecular marker for examining genetic diversity in mycobacteria, depends on pre-existing sequence knowledge and often demands significant DNA quantities. It utilizes targeted PCR genotyping for focused studies and microarray technology for extensive genomic screenings. While microarray technology provides thorough genomic insights, it has discernible limitations. For instance, some platforms cannot detect deletions smaller than 350 bp, potentially compromising the sensitivity of NTM infection identification (108). This limitation underscores the need for integrating or developing more refined molecular diagnostic tools capable of detecting smaller genetic variations. Furthermore, the challenge of cross-hybridization among similar sequences necessitates the optimization of probe design and hybridization conditions, particularly for the non-repetitive segments of bacterial genomes, to enhance the method’s applicability and accuracy in distinguishing between NTM subtypes. LSP analysis has been employed to identify differences between various subtypes within the MAC and *M. abscessus* (109).

Whole genome sequencing (WGS) represents a novel and highly precise method for identifying and characterizing various species of mycobacteria. Offering superior resolution compared to techniques such as targeted PCR genotyping and microarray analysis, WGS enables the differentiation of NTM subspecies down to their specific evolutionary branches, while also providing comprehensive genomic information. WGS provides unparalleled accuracy and precision in identifying genetic variances between strains by detecting almost all markers utilized in the genotyping methods mentioned previously. Its utility spans from offering comprehensive data at the global (population-wide), local (community), and individual (single patient) levels to yielding profound insights into the pathogens. It facilitates the precise identification of new NTM species and aids in predicting the virulence genes of NTM, laying a foundation for targeted therapy (50, 51). For example, WGS has successfully distinguished between closely related strains of *M. ulcerans* and *M. marinum*, providing insights into their evolutionary paths and virulence factors. Furthermore, its application in the study of MABC outbreaks has revealed specific transmission chains, highlighting its potential in public health surveillance and response (110, 111). Large-scale studies have validated the efficacy of WGS in mapping outbreaks and elucidating transmission pathways (112, 113). Compared with WGS, traditional methods such as PFGE and RFLP analysis often result in imprecise clustering, potentially missing fine-scale genetic variations critical for accurate pathogen identification and outbreak tracking. Furthermore, WGS has the capability to forecast undetected NTM species, thus facilitating the provision of timely and suitable interventions for emerging infections (114–117).

### 4.3 NGS

In recent years, NGS has significantly advanced our ability to harness comprehensive genetic information, especially via WGS. These technologies excel in pathogen detection with notable specificity, even amidst complex or sparse datasets, as demonstrated by metagenomic DNA analysis. Metagenomic NGS (mNGS) emerges as a superior, high-throughput method for DNA/RNA sequencing, outperforming traditional Sanger sequencing by offering quicker processing, reduced costs, and enhanced sensitivity. NGS markedly accelerates the breadth of genomic or transcriptomic analyses, substantially boosting sequencing speed and efficiency (118). This enhanced depth and coverage are particularly valuable in NTM infection identification, as they facilitate the detection of low-frequency mutations or variants that might be missed by other methods (119). In bacterial evolution analysis, NGS shows superior advantages, including applications in phylogenetic analysis, speculation on species origins, and predicting microbial communities (120–122). NGS encompasses diverse approaches, including mNGS and targeted NGS (tNGS), each offering distinct advantages and applications across various contexts.

mNGS represents a broadly unbiased sequencing technique, capable of sequencing the genomes of a wide array of microbes present in a sample, although its efficacy can be influenced by sample quality and sequencing depth. This approach offers significant advantages for organisms like NTM, which are notoriously difficult to culture and identify. Crucially, mNGS eliminates the need for prior knowledge of the microbial composition within a sample, allowing for direct DNA extraction and sequencing from the specimen. While circumventing the limitations and biases inherent to traditional culturing methods, it’s important to acknowledge scenarios where traditional methods may still offer valuable insights, particularly in understanding microbial growth characteristics and antibiotic susceptibility. mNGS facilitates the detection of microbial nucleic acids across a variety of specimen types and enables rapid identification of multiple pathogens, with numerous cases of NTM infections being diagnosed through this method (50, 52, 53).

Moreover, mNGS is adept at detecting low-abundance microbes, including NTM, making it essential for a comprehensive understanding of microbial communities within samples. mNGS exhibits the capability to uncover novel or hitherto unrecognized microbial species. In a study by Dougherty et al. (123), the utility of metagenomic analysis in identifying mixed infections was further corroborated. Their study underscored the prowess of shotgun metagenomics, not only for sequencing DNA from uncultured samples, but also for elevating mixed infection detection via target-specific amplification. This highlights its capability to refine microbial identification techniques. Thus, metagenomic analysis could signify a breakthrough in diagnostics, especially for multidrug-resistant (MDR) and extensively drug-resistant (XDR) strains.

tNGS is a high-throughput sequencing method focused on specific genes or genomic regions, playing a pivotal role in the identification and study of NTM. This technique begins with the alignment of sequenced DNA against NTM sequences from curated reference databases, a critical step that enables the precise identification of specific microbial species and subspecies within the sample. Subsequently, by analyzing sequence variations, tNGS can elucidate critical information such as the NTM subtype, virulence factors, and resistance genes (124, 125). By focusing sequencing efforts on targeted genes or genomic regions of interest, tNGS not only reduces costs and turnaround times compared to WGS but also enables the rapid identification of resistance genes and virulence factors, crucial for tailoring treatment strategies in NTM infections. This streamlined focus makes tNGS an invaluable tool in the nuanced field of NTM research, offering precise insights into microbial genetics with efficiency (126, 127). While tNGS offers significant advantages in terms of cost and speed, it’s important to acknowledge that by focusing only on predetermined genomic regions, it may miss novel or unexpected variants outside these regions, potentially limiting the comprehensive understanding of microbial genome complexity.

## 5 Exploring molecular diagnostics for NTM infections: beyond pathogen identification to advances in DST

Merely identifying the pathogen is insufficient for clinical satisfaction. Once the species of mycobacteria in a clinical sample is identified, the crucial next step is to determine its drug susceptibility to devise an appropriate treatment strategy. Inherent resistance, characteristic of certain NTM species, guides initial antibiotic choices, whereas acquired resistance, emerging during treatment, necessitates ongoing monitoring and potentially adjusting therapeutic strategies. While studies on MTB have laid the foundation for understanding antibiotic resistance mechanisms, the genetic and phenotypic diversity among NTM species necessitates targeted research to uncover specific resistance patterns and develop effective treatments. Increasing reports indicate that antibiotics previously effective, including macrolides, fluoroquinolones, and aminoglycosides, are now facing resistance, an issue particularly prevalent and posing a higher risk in slow-growing NTM, exacerbated by the extensive use of these antibiotics in both clinical and animal husbandry. Settings, leading to increased selection pressure for resistant strains (128, 129).

Managing NTM infections is notably challenging, largely owing to two main factors. First, the relationship between *in vitro* DST outcomes and clinical success is not consistently direct. This inconsistency can be attributed to factors such as the complex biology of NTM species and variability in patient responses to treatment. For example, DST results for *M. abscessus* and *Mycobacterium simiae* (*M. simiae*) have a weak correlation with clinical efficacy, in contrast to species such as *M. kansasii*, *M. marinum*, and *M. fortuitum*, where there is a stronger alignment with treatment success (130, 131). This variation in correlation may be due to differences in the mechanisms of antibiotic resistance and pathogenicity among these species, affecting the predictability of treatment outcomes based on DST alone. Secondly, clinical case management ranges from no treatment to requiring a multidrug regimen. The decision to opt for no treatment, monitor, or pursue aggressive multidrug regimens is guided by a comprehensive evaluation of the specific NTM species involved, infection severity, and individual patient health considerations, highlighting the need for a personalized approach to treatment. Thus, DST, aligned with Clinical and Laboratory Standards Institute (CLSI) guidelines, is critical for crafting optimal treatment strategies. Adhering to CLSI guidelines, which are regularly updated to reflect emerging data on NTM behavior and antibiotic resistance, ensures that DST is applied effectively to inform treatment strategies, underscoring the dynamic nature of NTM management. Identification of clinically significant antibiotic resistance genes (ARGs) enables the prediction of pathogen resistance.

The CLSI guidelines were revised and expanded in 2018, establishing broth microdilution as the most common laboratory method for DST to determine the minimum inhibitory concentration (MIC) (132). Traditional culture-dependent approaches for DST, prevalent in clinical microbiology laboratories, suffer from drawbacks including lengthy processing times, bias towards certain microbial communities, and contamination risks from overgrowth. Recent studies have elucidated the intrinsic and acquired resistance mechanisms of mycobacteria to classes of antibiotics including macrolides, aminoglycosides, oxazolidinones (such as linezolid), riminophenazines (like clofazimine), and di-substituted diazepanes (such as bedaquiline), paving the way for the application of molecular diagnostic techniques in DST (133). Notably, molecular diagnostics can also detect resistance heterogeneity, identifying resistant subpopulations within drug-sensitive mycobacterial communities (134, 135). The Comprehensive Antibiotic Resistance Database (CARD) (https://card.mcmaster.ca) aggregates well-documented, peer-reviewed resistance determinants alongside their corresponding antibiotics. Utilizing CARD facilitates the identification of genes and mutations associated with NTM resistance, enabling comparison of these sequences against WGS and NGS data. Regrettably, no existing database perfectly correlates NTM resistance predictions from WGS data with DST outcomes, reinforcing the need for refined bioinformatic tools and databases. Table 2 showcases a meticulously compiled inventory of NTM-relevant mutations and genes, including their reference data, sourced from the CARD (136–150). Table 3 outlines the comparison of DST and WGS in antibiotic susceptibility identification for NTM.

**Table 2:** Mutated genes associated with NTM and their mutation details retrieved from the CARD database.

NTM species	AMR gene family	CARD short name	Drug class	Resistance mechanism	References
*M. intracellulare*	23S rRNA with mutation conferring resistance to azithromycin	*Mint_23S_AZM*	Macrolide	antibiotic target alteration	(134)
	23S rRNA with mutation conferring resistance to clarithromycin	*Mint_23S_CLR*	Macrolide	antibiotic target alteration	(134)
*M. avium*	23S rRNA with mutation conferring resistance to clarithromycin	*Mavi_23S_CLR*	Macrolide	antibiotic target alteration	(135)
	Fluoroquinolone resistant *gyrA*	*Mavi_gyrA_FLO*	Fluoroquinolone	antibiotic target alteration	(136)
*M. kansasii*	23S rRNA with mutation conferring resistance to clarithromycin	*Mkan_23S_CLR*	Macrolide	antibiotic target alteration	(137)
*M. abscessus*	Antibiotic resistant ATP synthase	*Mabs_atpE_BDQ*	Diarylquinoline	Antibiotic target alteration	(138)
	16S rRNA mutation conferring resistance to kanamycin	*Mabs_16S_KAN*	Aminoglycoside	Antibiotic target alteration	(139)
	16S rRNA mutation conferring resistance to neomycin	*Mabs_16S_NEO*	Aminoglycoside	Antibiotic target alteration	(140)
	16S rRNA mutation conferring resistance to gentamicin	*Mabs_16S_GEN*	Aminoglycoside	Antibiotic target alteration	(139)
	16S rRNA mutation conferring resistance to tobramycin	*Mabs_16S_TOB*	Aminoglycoside	Antibiotic target alteration	(139)
	23S rRNA with mutation conferring resistance to clarithromycin	*Mabs_23S_CLR*	Macrolide	Antibiotic target alteration	(141, 142)
	16S rRNA mutation conferring resistance to amikacin	*Mabs_16S_AMK*	Aminoglycoside	Antibiotic target alteration	(141, 142)
*M. chelonae*	16S rRNA mutation conferring resistance to kanamycin A	*Mche_16S_KAN*	Aminoglycoside	Antibiotic target alteration	(140)
	16S rRNA mutation conferring resistance to neomycin	*Mche_16S_NEO*	Aminoglycoside	Antibiotic target alteration	(140)
	16S rRNA mutation conferring resistance to gentamicin C	*Mche_16S_GENC*	Aminoglycoside	Antibiotic target alteration	(140)
	16S rRNA mutation conferring resistance to tobramycin	*Mche_16S_TOB*	Aminoglycoside	Antibiotic target alteration	(140)
	23S rRNA with mutation conferring resistance to clarithromycin	*Mche_23S_CLR*	Macrolide	Antibiotic target alteration	(143)
	16S rRNA mutation conferring resistance to amikacin	*Mche_16S_AMK*	Aminoglycoside	Antibiotic target alteration	(140)
	23S rRNA with mutation conferring resistance to clarithromycin	*Msme_23S_CLR*	Macrolide	Antibiotic target alteration	(144)
*M. smegmatis*	23S rRNA with mutation conferring resistance to clarithromycin	*Msme_23S_CLR*	Macrolide	Antibiotic target alteration	(144)
	16 s rRNA with mutation conferring resistance to aminoglycoside antibiotics	*Msme_16rrsA_HGM*	Aminoglycoside	Antibiotic target alteration	(145)
	16 s rRNA with mutation conferring resistance to aminoglycoside antibiotics	*Msme_16rrsB_HGM*	Aminoglycoside	Antibiotic target alteration	(145)
	Antibiotic resistant *ndh*	*Msme_ndh_INH*	Isoniazid-like	Antibiotic target alteration	(146)
	16 s rRNA with mutation conferring resistance to aminoglycoside antibiotics	*Msme_16rrsB_STR*	Aminoglycoside	Antibiotic target alteration	(147)
	16 s rRNA with mutation conferring resistance to peptide antibiotics	*Msme_16rrsB_VIO*	Peptide	Antibiotic target alteration	(148)
	16 s rRNA with mutation conferring resistance to aminoglycoside antibiotics	*Msme_16rrsB_KAN*	Aminoglycoside	Antibiotic target alteration	(148)
	16 s rRNA with mutation conferring resistance to aminoglycoside antibiotics	*Msme_16rrsA_KAN*	Aminoglycoside	Antibiotic target alteration	(148)
	16 s rRNA with mutation conferring resistance to aminoglycoside antibiotics	*Msme_16rrsA_NEO*	Aminoglycoside	Antibiotic target alteration	(148)
	16 s rRNA with mutation conferring resistance to aminoglycoside antibiotics	*Msme_16rrsB_NEO*	Aminoglycoside	Antibiotic target alteration	(148)

AMR, antimicrobial resistance; CARD, the comprehensive antibiotic resistance database.

**Table 3:** Comparison of DST and WGS in antibiotic susceptibility identification for NTM.

Methods	Advantages	Limitations	Optimizing strategy
DST	Reliable and valid. Cost-effective and straightforward. Provides clear results and direct therapeutic guidance based on guidelines.	Extended duration, complex calibration, and tough quality control cause variable outcomes. Manual handing causes inconsistencies. Weak link between lab tests and clinical efficacy, with poor detail on drug resistance profiles.	Develop new culturing techniques and media to: Broaden testing scope. Reduce turnaround times. mplement standardized testing and quality control protocols.
WGS	High-resolution methods identify more mutations quickly. Eliminate need for existing resistance databases. Enhance data sharing and comparative analysis.	Lack of precise antimicrobial resistance databases reduces accuracy. High costs and technical requirements demand advanced bioinformatics and tools. DNA must be extracted from positive cultures,not directly from primary samples.	Establish extensive databases and sophisticated tools. Adopt standardized testing and quality control protocols. Consolidate databases to improve precision in medical research.

DST, drug sensitivity testing; WGS, whole genome sequencing.

Given the predominance of macrolide antibiotics as the first-line treatment for most NTM infections, the detection of resistance to these drugs is particularly critical. Macrolide resistance mechanisms involve the methylation of 23S rRNA by the erythromycin ribosome methylation (*erm*) gene, preventing macrolide binding to its ribosomal target. Additionally, mutations in the 23S rRNA itself can render macrolides ineffective, a scenario relatively common in mycobacteria due to their limited number of rRNA operons. A single mutation in any of these operons can significantly alter the ribosomal structure, inhibiting macrolide binding (151–153). Rifampin remains among the preferred treatments for most NTM infections, generally linked to mutations in its target, the *rpoB* gene, which encodes the β-subunit of RNA polymerase. Studies suggested rifampin preferentially inhibits one of the rpoB promoters (Promoter I), leading to increased expression from the second promoter (Promoter II) and promoting the growth of resistant strains (154). Recent research indicates additional mechanisms might be involved. There are reported cases of rifampin resistance in both MAC and *M. kansasii* (155, 156). *M. abscessus* resistance constitutes a formidable clinical hurdle, as evidenced by multiple DST studies demonstrating resistance across several antibiotic classes, including macrolides, quinolones, and aminoglycosides. Notably, intrinsic high-level resistance has been predominantly associated with established mutations in the *embB* gene, conferring resistance to ethambutol, and the *gyrA* gene, conferring resistance to fluoroquinolones (157). Additionally, intrinsic resistance to rifampin in *M. abscessus* is due to mutations in the *rpoB* gene and the presence of the *MAB_0591* gene, which is known to contribute to rifampin resistance processes (158). Another mechanism of intrinsic resistance is the overexpression of efflux pumps, contributing to resistance against bedaquiline and clofazimine (159). Chen et al. (160) conducted WGS on clofazimine-resistant *M. abscessus* strains, revealing several significant mutations in the *MAB_2299c*, *MAB_1483*, and *MAB_0540* genes, which were found to play crucial roles in conferring drug resistance. The identification of the *erm41* gene and its pivotal role in macrolide resistance has facilitated the critical differentiation of three distinct species within the MABC: *M. abscessus* subsp. abscessus (MABSa), *M. abscessus* subsp. massiliense (MABSm), and *M. abscessus* subsp. bolletii (MABSb). Prior to this discovery, the American Thoracic Society/Infectious Disease Society of America (ATS/IDSA) guidelines on NTM infections did not reflect the central influence of the *erm41* gene on macrolide susceptibility. This oversight has significant clinical implications, as the majority of MABC isolates exhibit a functional erm41 gene, correlating with poor treatment outcomes in patients (161). In MABSa, a T/C polymorphism at position 28 of the *erm41* gene determines inducible macrolide resistance (28 T) or susceptibility (T28C). While most MABSa and MABSb strains exhibit inducible macrolide resistance, those with the T28C substitution are sensitive due to the loss of *erm41* functionality. Conversely, MABSm contains a large deletion in the *erm41* gene, resulting in nonfunctional *erm41* and macrolide susceptibility (162, 163). The commercialized GenoType NTM-DR kits (Hain Life science, Germany) enables rapid identification of *M. abscessus* subspecies and concurrent detection of resistance to macrolides and aminoglycosides. Studies have demonstrated the test’s high sensitivity in detecting acquired resistance, underscoring its utility in the molecular diagnostics landscape for NTM infections (164, 165).

The adoption of WGS has markedly advanced our comprehension of NTM antibiotic resistance, thus improving treatment strategies. In a significant validation of WGS’s precision, Wetzstein et al. (166) demonstrated complete concordance between WGS forecasting of *M. abscessus* resistance to macrolides and aminoglycosides and results from both GenoType NTM-DR kits and phenotypic DST. Lipworth et al. (167) leveraged sequencing data from *M. abscessus* to identify novel mutations in the *erm* gene and *rrs* gene linked to macrolide resistance, mutations not covered by traditional genotyping, raising the possibility of false-negative outcomes. Yoshida et al. (168) developed a WGS-based diagnostic approach, incorporating DNA chromatography and PCR, to distinguish macrolide-resistant and macrolide-sensitive *M. abscessus* subspecies, including *M. massiliense*, *M. abscessus*, and *M. bolletii*, achieving a 99.7% concordance with conventional DST findings. With a promising start, Realegeno et al. (169) have formulated a WGS assay aimed at accurately predicting *M. abscessus* resistance to clarithromycin and amikacin, achieving an impressive 100% accuracy when compared to phenotypic results. Yet, it should be noted that before wide clinical application of such a WGS-based prediction method, extensive validation against an array of drugs and broader NTM species is imperative. NGS, encompassing both mNGS and tNGS, is increasingly valued for its potential in detecting drug resistance genes in NTM. tNGS, in particular, sequences specific genes or genomic regions, offering higher specificity in detecting target genes compared to mNGS (126). Due to the highly conserved DNA sequences within the Mycobacterium and limited nucleotide sequence data, mNGS often fails to accurately identify species within the Mycobacterium or detect multiple drug-resistant mutations (170). tNGS combines multiplex PCR amplification with sequencing to rapidly capture diverse drug resistance gene sequences, thus advancing the molecular diagnosis of NTM resistance (124, 125).

## 6 Clinical applications of molecular diagnostic techniques in the diagnosis of NTM

In contemporary clinical practice, advances in molecular biology have revolutionized the diagnosis of NTM infections. These methodologies offer rapid, accurate, and comprehensive detection capabilities for NTM species identification and genotyping, along with critical insights into antimicrobial resistance and susceptibility. The systematic standardization, which ensures consistency and reliability across different laboratories, and a modular approach that allows for the tailored combination of diagnostic tests, together with rigorous quality control measures, significantly enhance the efficacy and reliability of clinical diagnostics and thereby inform more precise therapeutic strategies.

**Figure 1:**
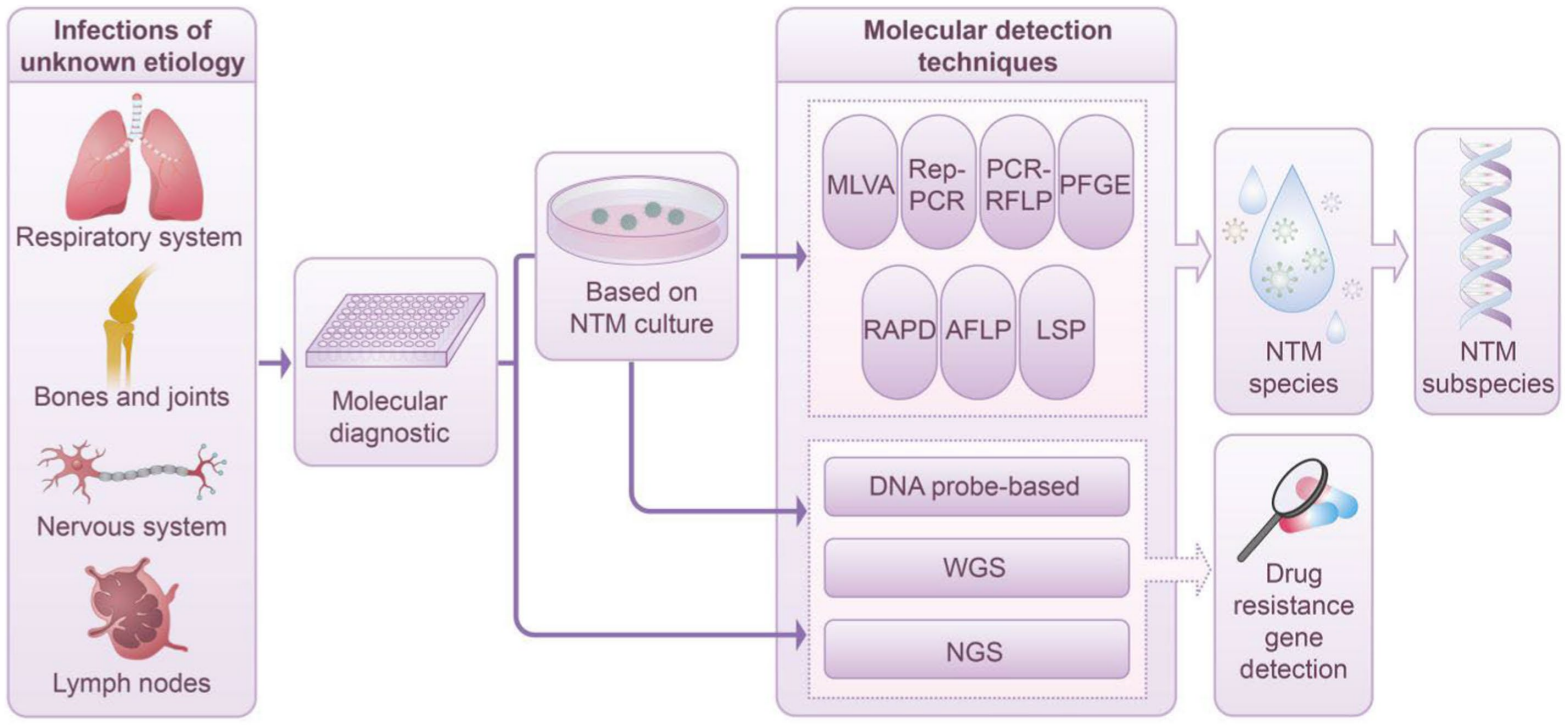
Diagnostic workflow based on molecular technology in NTM infection management. This schematic depicts the integration of advanced molecular techniques within the clinical framework for the diagnosis of NTM infections. Techniques such as MLVA, Rep-PCR, PCR-RFLP, PFGE, RAPD, AFLP, LSP, and DNA probe-based techniques, along with WGS and NGS, facilitate the detailed characterization of NTM species and their subspecies. The process entails isolating and culturing a singular NTM colony from a clinical specimen, followed by its purification. Notably, cutting-edge methodologies such as DNA probe-based techniques, WGS, and NGS enable direct application to clinical samples, thereby circumventing the conventional culture step. Upon completing the identification of NTM species and subspecies, tools such as DNA probe-based techniques, WGS, and NGS are employed to comprehensively evaluate NTM resistance genes. This approach significantly enriches our understanding of the mechanisms underlying microbial resistance.

Numerous molecular techniques are applicable for species identification and genotyping of NTM, each offering distinct advantages and specific applications in clinical settings. Techniques such as MLVA, MIRU-VNTR, Rep-PCR, RFLP, PFGE, RAPD, AFLP, and LSP are primarily employed for NTM subspecies in clinical diagnostics. Conversely, methods such as hybridization probes, WGS, and NGS provide significant advantages in identifying drug resistance genes. This capability is particularly critical in anticipating NTM drug resistance, especially in the absence of available DST. Figure 1 depicts the application and workflow of molecular diagnostic techniques in NTM species and subspecies identification.

## 7 Revolutionizing NTM classification and phylogeny with molecular diagnostic

The delineation and phylogenetic affiliations of NTM remain critical challenges due to their diverse genetic backgrounds and widespread environmental presence, which complicate clinical identification and treatment. Conventional methodologies, primarily relying on phenotypic characteristics and limited genetic markers, often lead to inconsistent and conflicting taxonomic identifications when different tools analyze the same environmental samples. The NTM taxonomy is dynamic, with phylogenetic updates substantially influencing the terminology across various fields. Advancements in molecular diagnostics, particularly through 16S rRNA gene amplification and sequencing, have unveiled extensive microbial diversity and intricate interrelations (171, 172). Nonetheless, exclusive reliance on 16S rRNA gene-based taxonomy presents significant shortcomings, such as inadequate resolution at and below the genus level, primer mismatches, and the potential distortion of phylogenetic relationships owing to chimeric sequence formation during PCR, which may impact the integrity of phylogenetic trees (173, 174). Thus, there’s a necessity for genomic data, beyond the 16S rRNA gene sequences, for accurate species classification and matching (175). Analyses of metagenomes and the construction of phylogenetic trees using single-copy, vertically transmitted protein sequences provide enhanced resolution compared to those derived from a single phylogenetic marker gene, such as 16S rRNA. These approaches are increasingly recommended for taxonomic reference (176, 177). The advent of the molecular diagnostics era, marked by the application of technologies such as NGS, including WGS and mNGS, has not only clarified NTM genomes but also provided a more accurate and comprehensive understanding of their phylogenetic relationships, thereby overcoming the limitations of previous methodologies. Genomic data enables the construction of more robust, comprehensive phylogenetic trees with higher resolution. A comprehensive review of the literature was undertaken, focusing on instances where molecular methodologies have been utilized to construct phylogenetic and evolutionary analyses of NTM, as detailed in Table 4 (178–185).

**Table 4:** Published data on studies involving molecular diagnostic techniques in constructing phylogenetic and evolutionary of NTM.

Author	Year	Bacteria	Methodology	Outcome	References
Fedrizzi et al.	2017	41 NTM type strains	NGS	Found clearly distinct evolutionary pathways for slow and rapidly growing mycobacteria in agreement with the pre-NGS era phylogeny.	(176)
Tortoli et al.	2017	144 NTM type strains	NGS	Refined the phylogeny of NTM by expanding the analysis to 88 strains	(177)
Gupta et al.	2018	150 NTM type strains	WGS	Phylogenetic trees were constructed for 150 members of the genus mycobacterium	(178)
Chew et al.	2021	*M. abscessus*	WGS	The risk of patient-to-patient transmission appears to be largely limited to the vulnerable CF population, indicating infection from environmental sources remains more common than human-to-human transmission	(179)
Das et al.	2018	*M. marinum*	WGS	*M. marinum* should be divided into two different clusters, the “M”- and the “Aronson”-type	(180)
Uchiya et al.	2017	*M. avium*	WGS	MAH had the highest degree of sequence variability among the subspecies, and MAH strains isolated in Japan and those isolated abroad possessed distinct phylogenetic features.	(181)
Stinear et al.	2008	*M. marinum*	WGS	Comparisons with the more distantly related *M. avium* subspecies paratuberculosis and *M. smegmatis* reveal how an ancestral generalist mycobacterium evolved into MTB and *M. marinum*	(182)
Qi et al.	2009	*M. ulcerans*	NGS	Offered significant insight into the evolution of *M. ulcerans* and provide a comprehensive report on genetic diversity within a highly clonal *M. ulcerans* population from a Buruli ulcer endemic region.	(183)
Matsumoto et al.	2019	175 NTM type strains	NGS	Expanded the available genomic data to 175 NTM species and redefined their subgenus classification	(82)

NGS, next-generation sequencing; WGS, whole genome sequencing; CF, cystic fibrosis; MAH, *Mycobacterium avium* subsp. hominissuis; MTB, *Mycobacterium tuberculosis*.

## 8 Integration of molecular diagnostic technologies with novel pathogen identification techniques

Matrix-assisted laser desorption/ionization time-of-flight mass spectrometry (MALDI-TOF MS) offers significant advantages for biomolecular analysis (186). It has proven effective for identifying pathogenic microorganisms and their resistance patterns, demonstrating specificity and sensitivity superior to Sanger sequencing, with detection limits on par with next-generation sequencing (187–191). While the identification of bacterial isolates, including NTM, is well established using MALDI-TOF MS, the technology is optimized for pure cultures. Challenges arise with mixed Mycobacterium cultures, where molecular methods are necessary to identify multiple NTM species in respiratory specimens. Despite its utility, differentiation of closely related species, such as subspecies of *M. abscessus*, poses difficulties. Literature reports occasional misidentifications by MALDI-TOF MS, primarily among closely related species or those within the same mycobacterial complex (192). Although some studies have explored subspecies identification through protein peak analysis via MALDI-TOF MS, a consensus on the optimal approach remains elusive (193–195). Recent years have seen the adoption of advanced data analysis techniques, including machine learning, which extend beyond simple species identification to provide more comprehensive insights (196). While MALDI-TOF MS has demonstrated efficacy in DST, it has yet to gain global consensus (197). In summary, MALDI-TOF MS offers rapid, sensitive, accurate, and cost-effective detection with high resolution and minimal sample requirements, enabling precise identification of NTM. Nevertheless, for complex NTM infections, integration with newer molecular diagnostic technologies is essential for accurate identification.

High-performance liquid chromatography (HPLC), developed in the mid-1960s within the chemical sciences, has been extensively applied across inorganic, organic, and biochemical disciplines (198). In the realm of NTM species identification, HPLC facilitates the analysis of mycobacterial components like mycolic acids, offering a faster alternative to traditional, time-intensive identification methods (199). While HPLC enhances species-level differentiation over biochemical tests, its effectiveness is limited by its inability to consistently discern clinically significant NTM species (200). Moreover, the proliferation of similar HPLC chromatograms among various species has diminished its utility as a standalone diagnostic tool (201). Consequently, for robust and accurate NTM species identification and typing, HPLC should be integrated with other molecular diagnostic techniques.

## 9 Limits and future challenges in molecular diagnostic for NTM identification

Despite the benefits of molecular diagnostics for NTM infection identification, the literature identifies key limitations and challenges. The lack of standardized protocols results in operational and data processing variability, impacting comparability and reproducibility across labs. Additionally, the necessity for high-quality samples and the variability in sample sources complicate standardizing pretreatment procedures, affecting detection accuracy and reliability. Furthermore, the elevated costs and complexity of certain molecular methods limit their broad clinical adoption, underscoring the need for more affordable and straightforward technologies to enhance their practicality and acceptance in clinical settings.

In upcoming research, molecular diagnostics will focus on key areas. Microfluidic technologies, known for their high throughput, sensitivity, and cost-effectiveness, are expected to revolutionize NTM detection by enabling automated sample processing and analysis. Additionally, integrating artificial intelligence (AI) and machine learning (ML) shows promise in extracting insights from extensive molecular data, aiding in precise diagnostic and treatment decisions. Nucleic acid mass spectrometry (NAMS) has gained prominence, with clinical laboratories increasingly adopting these techniques, poised to become standard in routine lab settings, offering potential for NTM infection detection advancements (202). Advances in genome editing technologies, like CRISPR/Cas9, offer opportunities for precise NTM genome editing, enhancing understanding of pathogenic mechanisms, drug responses, and resistance (203). These technological advancements will deepen comprehension of NTM diseases and drive diagnostic and therapeutic methodologies forward.

## 10 Conclusion

Molecular diagnostics, notably through WGS and NGS, have revolutionized NTM infection research, providing efficient, precise tools for species identification and drug sensitivity prediction. These methods have exceeded traditional limitations, offering fresh insights into NTM treatment approaches. Yet, challenges remain in studying less common NTM species, highlighting the need for technique refinement and a deeper understanding of NTM diversity.

In summary, the significant influence of molecular diagnostics on NTM identification is undeniable. As research progresses and technological refinement continues, there exists a potential for pioneering discoveries that will elucidate the complex biology of NTM and enable the development of more precise and effective diagnostic and therapeutic strategies for conditions related to NTM.

## Author contributions

HZ: Funding acquisition, Methodology, Resources, Writing – original draft. TM: Conceptualization, Writing – original draft. DL: Supervision, Validation, Writing – review & editing. MX: Conceptualization, Writing – original draft. YA: Investigation, Writing – review & editing. LL: Methodology, Project administration, Resources, Supervision, Writing – original draft, Writing – review & editing.
